# Biological roles and potential clinical values of circular RNAs in gastrointestinal malignancies

**DOI:** 10.20892/j.issn.2095-3941.2020.0348

**Published:** 2021-06-15

**Authors:** Xueping Tao, Yongfu Shao, Jianing Yan, Liyang Yang, Qihua Ye, Qingling Wang, Rongdan Lu, Junming Guo

**Affiliations:** 1 Department of Biochemistry and Molecular Biology and Zhejiang Key Laboratory of Pathophysiology, Ningbo University School of Medicine, Ningbo 315211, China; 2 Department of Gastroenterology, the Affiliated Hospital of Medical School of Ningbo University, Ningbo 315020, China

**Keywords:** Circular RNA, gastrointestinal malignancies, biological function, clinical value, digestive system

## Abstract

Circular RNAs (circRNAs), a class of endogenous RNA molecules, are produced by alternative splicing of precursor RNA and are covalently linked at the 5′ and 3′ ends. Recent studies have revealed that dysregulated circRNAs are closely related to the occurrence and progression of gastrointestinal malignancies. Accumulating evidence indicates that circRNAs, including circPVT1, circLARP4, circ-SFMBT2, cir-ITCH, circRNA_100782, circ_100395, circ-DONSON, hsa_circ_0001368, circNRIP1, circFAT1(e2), circCCDC66, circSMARCA5, circ-ZNF652, and circ_0030235 play important roles in the proliferation, differentiation, invasion, and metastasis of cancer cells through a variety of mechanisms, such as acting as microRNA sponges, interacting with RNA-binding proteins, regulating gene transcription and alternative splicing, and being translated into proteins. With the characteristics of high abundance, high stability, extensive functions, and certain tissue-, time- and disease-specific expressions, circRNAs are expected to provide novel perspectives for the diagnoses and treatments of gastrointestinal malignancies.

## Introduction

Malignant cancers pose a serious threat to human health. The latest evidence indicates that the incidence of cancers of the digestive system is over 30%, with more than 50 million new cases worldwide every year, ranking first among cancers of different body systems, and posing a major threat to human health^1^. Due to their high incidence, insidious onset, high degree of malignancy, and propensity for metastasis, gastrointestinal malignancies [such as gastric cancer (GC), colorectal cancer (CRC), hepatocellular carcinoma (HCC), pancreatic cancer, and esophageal cancer] are difficult to diagnose and treat early^2–5^.

Circular RNAs (circRNAs), a class of endogenous circular RNA molecules, are produced by alternative splicing of precursor RNAs, during which the 5′ and 3′ ends are covalently linked^6^. Accumulating studies have demonstrated that circRNAs play critical roles in the pathological processes of gastrointestinal malignancies, including tumorigenesis, development, and metastasis^6–8^. CircRNAs are considered to be important gene expression regulators. At the epigenetic, transcriptional, and posttranscriptional levels, circRNAs are involved in cancer cell proliferation, differentiation, invasion, and metastasis by sponging microRNA (miRNA), interacting with RNA-binding proteins (RBPs), regulating gene transcription and alternative splicing, and being translated into proteins^8–10^. Moreover, with the characteristics of high abundance, high stability, extensive functions, and certain tissue, time, and disease specificities, circRNAs are expected to serve as novel diagnostic biomarkers and new treatment targets for gastrointestinal malignancies^3,11,12^.

Hence, the relationships between circRNAs and gastrointestinal malignancies are an important research field. Here, we focus on the biological roles and potential value of circRNAs to provide new ideas for a better understanding of the pathogenesis of gastrointestinal malignancies, as well as new perspectives for the study of prevention and treatment strategies.

## Biogenesis

**Figure 1:**
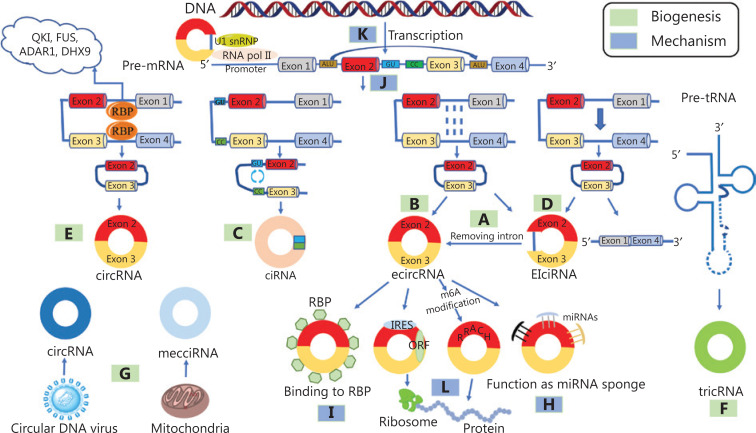
Biogenesis of circRNAs and the mechanisms of action of circRNAs. (A) Lariat-driven circularization (ecircRNA). (B) Intron-pairing-driven circularization (ecircRNA). (C) Circular intronic RNA. (D) Exon-intron-derived circRNA (EIciRNA). (E) RNA-binding protein (RBP)-driven circularization-derived circRNA. (F) The tRNA precursor-derived. (G) Other sources: fusion gene, circular DNA tumor virus, mitochondrial DNA-originated and mitochondria-encoded circular RNA (mecciRNA). (H) Functioning as miRNA sponges. (I) Interactions with RNA-binding proteins. (J) Involvement in alternative splicing regulation. (K) Regulation of parental gene transcription. (L) Protein-coding potential. QKI, Quaking; FUS, fused in sarcoma; ADAR1, adenosine to inosine acting on RNA enzyme 1; DHX9, DEAH-box helicase 9. IRES, internal ribosome entry site; ORF, open reading frame.

**Figure 2:**
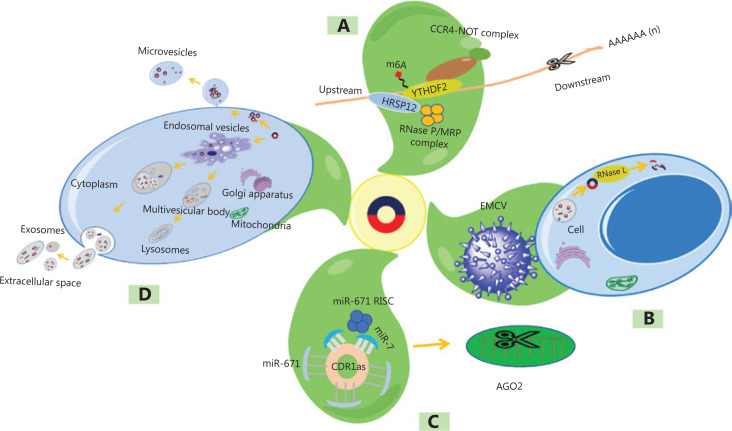
Clearance of circRNAs. (A) N6-methyladenosine-mediated degradation. (B) RNase L-mediated circRNA degradation after viral infection. (C) Argonaute protein 2 (AGO 2)-mediated degradation. (D) CircRNAs released in the form of extracellular vesicles. YTHDF2, YTH domain-containing family 2; HRSP12, heat-responsive protein 12; CCR4, carbon catabolite repression 4; MRP, multidrug resistance-associated protein; EMCV, encephalomyocarditis virus; RISC, RNA-induced silencing complex.

**Figure 3:**
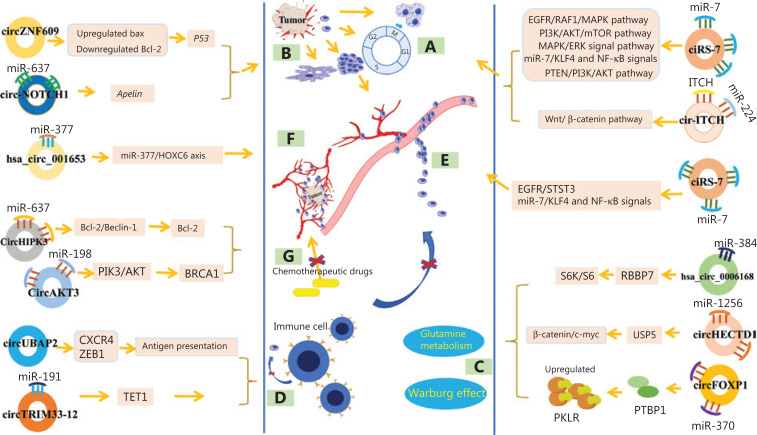
The relationships of circRNAs with gastrointestinal malignancies. (A) Cell proliferation, differentiation and cell cycle regulation. (B) Cell apoptosis regulation. (C) Cell metabolism regulation. (D) Immune escape. (E) Invasion and metastasis regulation. (F) Angiogenesis regulation. (G) Drug resistance regulation. EGFR, epidermal growth factor receptor; MAPK, mitogen-activated protein kinase; PI3K, phosphatidylinositol 3-kinase; AKT, protein kinase B; mTOR, mammalian target of rapamycin; MAPK, mitogen-activated protein kinase; ERK, extracellular regulated protein kinases; KLF4, Krüppel-like factor4; NF-κB, nuclear transcription factor-κB; PTEN, phosphatase and tensin homology deleted on chromosome ten; CXCR4, C-X-C chemokine receptor type 4; ZEB1, zinc finger E-box binding protein 1; PTBP1, polypyrimidine tract-binding protein 1; PKLR, L-type pyruvate kinase; USP5, ubiquitin-specific peptidase 5; BRCA1, breast cancer type 1; bcl-2, B-cell lymphoma-2; HOXC6, homeobox C6; TET1, tet methylcytosine dioxygenases 1; S6K, S6 kinase; RBBP7, retinoblastoma-binding protein 7.

CircRNAs are mainly generated by the alternative splicing of a class of precursor RNAs and covalently linked to form a circular structure^8^. In eukaryotic cells, circRNAs can be classified into the following 6 main types based on their genomic origin: (1) exonic circular RNA (ecircRNA), which can be generated by intron-pairing-driven circularization (**Figure 1A**) and lariat-driven circularization (**Figure 1B**); (2) circular intronic RNA (ciRNA; **Figure 1C**); (3) exon-intron-derived circRNA (EIciRNA; **Figure 1D**); (4) RBP-driven circularization-derived circRNA (**Figure 1E**); (5) tRNA precursor derived circRNA (tricRNA; **Figure 1F**); and (6) circRNAs from other sources (**Figure 1G**).

### Lariat-driven circularization and intron-pairing-driven circularization of exonic circRNAs

Lariat-driven circularization is also known as the exon skipping mechanism. During transcription, mRNA precursors can partially fold, causing some exons to jump with the folding of the RNA to form a lariat structure, after which the introns within the lariat structure are removed to form an ecircRNA (**Figure 1A**). Kelly et al.^13^ confirmed that there were numerous circRNAs derived from lariat-driven circularization by first analyzing the relationship between the abundance of each exon in poly(A)+ mRNA and circularized mRNA, then plotting the relative retention rate of each exon in mature transcripts as a function of its circularization, and controlling for the decrease in exon abundance caused by the decrease in maternal gene expression.

In intron-pairing-driven circularization, two flanking introns have reverse complementary structures (RCMs), such as Alu repeats, and the pairing of flanking introns bring splice junction sites close to each other, forming a secondary structure that mediates the back-splicing process to form an ecircRNA (**Figure 1B**). For example, in the flanking regions of hsa_circ_POLR2A, there are several relatively short introns with an upstream reverse Alu sequence and two downstream forward Alu sequences, which form inverted repeated Alu pairs (IRAlus) with reverse complementary structures that mediate the circularization of hsa_circ_POLR2A^14^.

### Circular intronic RNAs

The circularization process of intronic RNA is driven by several short sequences located in specific introns, including the GU-enriched sequence near the 5′ end of the splice site and the C-rich sequence near the branch point (**Figure 1C**). The reverse complementary interactions between these short sequences ensure that they bind to form a lariat structure; then, the lariat is spliced by the spliceosome, which facilitates the formation of ciRNAs^15^.

### Exon-intron-derived circRNAs

In the process of reverse splicing of a class of circRNAs related to RNA polymerase II (pol II), while exons are circularized, some intron sequences are not removed but retained, forming EIciRNAs (**Figure 1D**). For example, circEIF3J is an atypical EIciRNA. It can bind to the U1 snRNP binding site in the retained introns to accelerate the transcription of the parental genes^16^.

### RBP-driven circularization-derived circRNAs

RBPs are involved in the circularization process. Some RBPs specifically bind to the flanking intron sequences of the transcript, bringing the donor sites and the acceptor sites close to each other and inducing exon circularization to form circRNAs (**Figure 1E**). For example, quaking-5 (QKI-5), an RBP, was initially recognized as a tumor suppressor. The latest evidence suggests that during the epithelial-mesenchymal transition (EMT), QKI-5 promotes exon circularization to form circRNA by binding to flanking intron sequences^17^.

### The tRNA precursor-derived circRNAs

In the process of metazoan pre-tRNA self-splicing, tRNA splicing endonuclease (TSEN) recognizes and cleaves the canonical bulge-helix-bulge (BHB) motif of the anticodon loop^18^. Then, the generated 2′,3′-cyclic phosphate groups and 5′-OH are linked by the RNA ligase RtcB to form tricRNA (**Figure 1F**). In a similar manner, eukaryotic pre-tRNA was discovered to form tricRNAs, which are mostly located in the cytoplasm and are highly conserved^19^.

### Other sources

Apart from the above mechanisms, there are other sources of circRNAs (**Figure 1G**). The flanking introns of the reverse splicing site have been confirmed to promote the circularization of a fusion gene to form a new type of circRNA. For example, F-circSR1 and F-circSR2 are derived from the SLC34A2-ROS1 fusion gene^20^. F-circEA is derived from the EML4-ALK fusion gene^21^. Additionally, circular DNA tumor virus and mitochondrial DNA produce circRNAs^22,23^.

## Biological characteristics

Generally, circRNAs are widely expressed and abundant in diverse cell types and organisms, with over 10-fold higher abundance than that of linear RNA in some tissues^24^. Due to the lack of free 3′ and 5′ ends, circRNAs are resistant to degradation by debranching enzymes and RNA exonucleases, and stably exist in the cytoplasm^25^. The half-life of exon-derived circRNAs is more than 48 hours, which is significantly longer than the 10 h half-life of most mRNAs^24,26^. In particular, circRNAs are expressed in certain tissue-, time- and disease-specific patterns, making them potential diagnostic markers and therapeutic targets for diseases^3,11,12^.

## Clearance of circRNAs

In eukaryotes, circRNAs are highly conserved, and their clearance mechanism has been revealed in recent years, which mainly occurs by the following three processes.

### N6-methyladenosine-mediated degradation

N6-methyladenosine (m6A) modification, widely found in eukaryotes, is a chemical modification that adds a methyl group to the sixth (N) position of adenine in RNA molecules^27^. The m6A recognition protein contains YTH domain-containing family 2 (YTHDF2), which can bind the target molecules^28^. By recruiting heat-responsive protein 12 (HRSP12), YTHDF2 then mediates the cleavage of target molecules by the RNase P/MRP complex (**Figure 2A**). In addition, ribonuclease L (RNase L) can also mediate circRNA degradation after viral infection (**Figure 2B**).

### Argonaute protein 2-mediated degradation

Argonaute protein 2 (AGO2), a member of the widely expressed Argonaute protein family, has a conserved structure and plays an important role in RNA interference^29^. Some studies have indicated that miR-671 can trigger AGO2 to mediate CDR1 involving degradation by binding to a highly conserved miRNA binding site in CDR1as^30,31^. Moreover, miR-7 can recruit the miR-671 RNA-induced silencing complex (RISC) or be retained in ciRS-7 (CDR1as) through other unknown mechanisms, thereby enhancing the clearance process (**Figure 2C**).

### Extracellular vesicle output

Extracellular vesicles, membrane structures surrounded by a lipid bilayer and secreted by living cells, contain components such as RNA and proteins. Lasda et al.^32^ found that cultured cells secreted exosomes that contained circRNAs. Moreover, they found that circRNAs were enriched far more than linear RNAs^32^. CircRNAs can be removed from cells by secreted vesicles (such as exosomes or microvesicles) (**Figure 2D**).

## Mechanisms of action of circRNAs

CircRNAs were once regarded as byproducts and did not receive much attention. Recently, the biological mechanisms of action of circRNAs have been extensively revealed. CircRNAs have been found to exert their biological effects by functioning as microRNA (miRNA) sponges (**Figure 1H**), binding with RBPs (**Figure 1I**), regulating the alternative splicing (**Figure 1J**) and transcription of parent genes (**Figure 1K**), and being translated into proteins (**Figure 1L**).

### Function as miRNA sponges

Circular RNA sponge for miR-7 (ciRs-7), also known as antisense to the cerebellar degeneration-related protein 1 transcript (CDR1as), is a typical circRNA^33^. Because ciRs-7 was discovered as acting as a sponge of miRNAs, increasing attention has been directed to the interaction between circRNAs and miRNAs. The structural basis for circRNAs in regulating downstream miRNAs involves the existence of seed sequences that target miRNAs by incomplete complementary base pairing. When a circRNA adsorbs a functional miRNA, the activity of the miRNA is reduced, and its regulatory effect on downstream target genes is inhibited. Most of the circRNAs found so far can act as miRNA sponges to exert their biological effects. For example, CDR1as, which contains over 70 miR-7 binding sites, can absorb a large amount of miR-7 by functioning as a miR-7 sponge, thus decreasing the regulatory function of miR-7 and indirectly inhibiting tumor progression, cell proliferation, and invasiveness in multiple cancers (including GC and CRC)^33,34^.

CircRNAs can directly regulate downstream target genes by acting as miRNA sponges, and can also directly or indirectly participate in signal transduction pathways in the same manner. For example, Zhang et al.^35^ found that by adsorbing miR-149-5p, upregulated circNRIP1 in GC increased the expression level of mammalian target of rapamycin (mTOR) to activate the AKT1/mTOR signaling pathway, thus accelerating energy anabolism and inhibiting autophagy-related catabolism of GC cells.

### Binding to RBPs

CircRNAs can bind to proteins and either directly function as protein sponges or indirectly regulate RNA. The resulting RNA-protein complex then regulates the interaction between RNA and RBPs^36^. In addition, RBPs involved in the process of RNA alternative splicing affect protein functions and posttranscriptional gene expressions^36,37^. For example, circSMARCA5 has been confirmed to regulate the mRNA splicing of vascular endothelial growth factor-A (VEGFA) by affecting the expression and function of serine/arginine rich splicing factor 1 (SRSF1), subsequently influencing the progress of glioblastoma^37^. By interacting with DEAD-box polypeptide 3 (DDX3), circ-CTNNB1 has been shown to transactivate transcription factor Yin Yang 1 (YY1), promoting the expression of genes related to the activation of β-catenin protein and affecting tumor progression^38^.

### Alternative splicing regulation

As a common process in eukaryotic organisms, alternative splicing is a major source of biological protein diversity. The latest evidence confirmed that circRNAs participate in the process of alternative splicing^39^. For example, it was reported that circMbL affected linear RNA synthesis and regulated the expression of related genes by competing with the linear splicing of classical pre-mRNA^40^. In addition, circ-UBR5 has been shown to participate in the RNA splicing process by binding to splicing regulatory factor quaking (QKI), NOVA alternative splicing regulatory factor1, and U1 small nuclear RNA^39^.

### Transcriptional regulation of parental genes

One of the most prominent biological roles of RNAs is the regulation of parental gene expression. Similarly, some circRNAs have been confirmed to have similar regulatory functions. These circRNAs act as transcriptional regulators to participate in the transcription of parental genes and exert indirect biological effects. For example, circ-ITCH is transcribed from its parental gene *ITCH* and then regulates *ITCH* gene transcription by acting as a sponge for miR-7, miR-17, and miR-214, thereby inhibiting the Wnt/β-catenin pathway and downregulating the expression of the proto-oncogene *c-myc* through the ubiquitin-mediated degradation of phosphorylated Dvl2^41^. Similarly, circITGA7 has been shown to upregulate the transcription of its host gene integrin alpha 7 (ITGA7) by inhibiting the transcription factor RAS-responsive element binding protein 1 *via* the Ras pathway^42^.

### Translation into proteins

#### CircRNAs containing internal ribosomal entry sites can be translated into proteins

CircRNAs do not have a free 5′ cap or 3′ poly (A) tail. Thus, they lack an effective initial structure to guide protein translation. However, by inserting a synthetic internal ribosome entry site (IRES) upstream of the initial codon of protein synthesis, circRNAs can be translated into proteins^43^. For example, circ-ZNF609 contains an open reading frame (ORF) sequence bounded by a start codon and a stop codon, and can be translated into proteins in a splicing-dependent manner^44^. In another study, Van Heesch et al.^45^ systematically analyzed the mechanism and pattern of RNA translation in cardiac tissues and successfully found many translation products of circRNAs and 40 translatable circRNAs produced from 39 genes, including the well-known CDR1as and the newly detected circCFLAR, circSLC8A1, circMYBPC3, and circRYR2.

#### N6-methyladenine modification promotes circRNA translation

The m6A modification, an internal RNA modification widely existing in eukaryotes, has been demonstrated to affect multiple stages of RNA metabolism, including mRNA localization, splicing, translation, and degradation, and is related to various pathophysiological processes. Yang et al.^46^ indicated that RRm6ACH (R = G or A; H = A, C, or U), a recognized m6A motif enriched in circRNAs, which promoted the initiation of protein translation from circRNAs and also directly affected the translation efficiency. It is worth noting that m6A-driven translation requires the involvement of the initiating factor, eukaryotic translation initiation factor 4 gamma 2, and m6A reader YTH N6-methyladenosine RNA-binding protein 3, which can be enhanced by methyl transferase METTL3/14, inhibited by demethylase FTO, and upregulated during heat shock^46^.

## Relationship of circRNAs with gastrointestinal malignancies

CircRNAs participate in gene regulation and cancer-related signaling pathways. Dysregulated circRNAs are only involved in pathological processes such as cancer cell proliferation, differentiation, apoptosis, invasion, metastasis and angiogenesis, and also affect cell metabolism and drug sensitivity. In **Table 1**, we summarize the function and clinical significance of cancer-related circRNAs in gastrointestinal malignancies.

**Table 1:** Relationship between circRNAs and gastrointestinal malignancies

Cancer type	circRNA	Level	miRNA sponged	Target/pathways	Functional phenotypes	Clinical significance	References
Esophageal cancer	hsa_circ_0006168	Up	miR-384	S6K/S6 pathway	Promote cell proliferation, migration, invasion, and glycolysis	Therapeutic target	^58^
	circ_100876	Down	/	MMP13	Regulate cell proliferation, cell cycle, migration, invasion, and EMT	Prognosis predictor and therapeutic target	^122^
	circ-SLC7A5	Up	/	/	/	Diagnostic and prognostic biomarker	^129^
	hsa_circ_0001946	Down	miRNA-7-5P	/	Inhibit cell proliferation, migration, and invasion	Diagnostic and prognostic biomarker	^109^
	circ-Foxo3	Down	miR-23a	PTEN	Inhibit cell growth, migration, and invasion	Therapeutic target	^138^
	cir-ITCH	Down	miR-23a	Wnt/β-catenin	Reduce cell viability, and arrests proliferation	Therapeutic target	^41^
	ciRS-7	Up	miR-7	miR-7/KLF4 and NF-κB signals	Promote cell migration and invasion	Therapeutic target	^85^
	circ_0000337	Up	miR-670-5p	/	Regulate cell proliferation, migration and invasion	Therapeutic target	^86^
	hsa_circ_0006168	Up	miR-100	mTOR	Promote cell proliferation, migration and invasion	Therapeutic target	^87^
	circUBAP2	Up	miR-422a	Rab10	Promote cell proliferation, migration and invasion	Therapeutic target	^88^
	hsa_circ_0004771	Up	miR-339-5p	CDC25A	Promoted cell proliferation	Diagnostic biomarker and prognosis predictor	^123^
Gastric cancer	circPVT1	Up	miR-125	E2F2	Promoted cell proliferation	Prognostic marker	^121^
	circLARP4	Down	miR-424-5P	LAST1	Promoted cell proliferation and invasion	Prognostic marker	^124^
	circPIP5K1A	Up	miR-671-5p	PI3K/AKT pathway	Regulates cell proliferation, invasion, migration and EMT process	Therapeutic target	^89^
	circHECTD1	Up	miR-1256	PI3K/AKT pathway	Regulates cell glutaminolysis, proliferation, migration, and invasion	Prognostic marker and therapeutic target	^76^
	circAKT3	Up	miR-198	PIK3R1	Promotes DNA damage repair and inhibits apoptosis	Prognostic marker and therapeutic target	^66^
	circYAP1	Down	miR-367-5p	miR-367-5p/p27 ^Kip1^ axis	Inhibited cell growth and invasion	Prognostic marker and therapeutic target	^125^
	circNRIP1	Up	miR-149-5p	AKT1/mTOR	Regulates cell proliferation, migration, invasion and the expression level of AKT1	Prognostic marker and therapeutic target	^35^
	circ_0008035	Up	miR-599	EIF4A1	Promote cell growth and repressed apoptosis and ferroptosis	Therapeutic target	^54^
	circPSMC3	Down	miR-296-5p	PTEN	Inhibit the tumorigenesis	Diagnostic and prognostic biomarker	^136^
	circPDSS1	Up	miR-186-5p	NEK2	Promote cell cycle and inhibit apoptosis	Therapeutic target	^57^
	circ_0027599	Down	miR-101	PHLDA1	Inhibit cell proliferation and metastasis	/	^141^
Hepatocellular carcinoma	circMTO1	Down	miR-9	p21	Regulates cell proliferation and invasion	Prognostic marker and therapeutic target	^126^
	circFBXO11	Up	miR-605	miR-605/FOXO3/ABCB1 axis	Promote cell proliferation, cell cycle progress and OXA resistance	Prognostic marker and therapeutic target	^134^
	circMAN2B2	Up	miR-217	MAPK1	Regulate cell proliferation	Therapeutic target	^139^
	circABCB10	Up	miR-670-3p	HMG20A	Regulate cell proliferation and invasion	Therapeutic target	^59^
	hsa_circ_0000092	Up	miR-338-3p	HN1	Regulate cell proliferation, migration, invasion and angiogenesis	Therapeutic target	^101^
	hsa_circ_0056836	Up	miR-766-3p	FOSL2	Regulate cell migration, proliferation and invasion	Therapeutic target	^140^
	circ_100395	Down	miR-1228	/	Regulate cell proliferation, apoptosis, EMT pathway and migration and invasion ability	Therapeutic target	^52^
	circ_0101432	Up	miR-1258 and miR-622	MAPK1	Inhibit cell apoptosis, promote cell proliferation, invasive ability	Therapeutic target	^68^
	circ_0000267	Up	miR-646	/	Promote cell growth, migration and invasion and attenuate cell apoptosis	Prognostic marker	^62^
	circSETD3	Down	miR-421	MAPK14	Inhibit cell proliferation	Prognostic marker	^4^
	circ_0078710	Up	MiR-31	HDAC and CDK2	Promote cell proliferation, migration, invasion and tumor growth	/	^93^
	circSLC3A2	Up	miR-490-3p	PPM1F	Promote cell proliferation and invasion	Prognostic marker	^60^
	circTRIM33-12	Down	miR-191	TET1	Inhibit cell proliferation, migration, invasion and immune evasion	Prognostic marker	^78^
	circRHOT1	Up	/	NR2F6	Promote cell growth, migration and invasion	Prognostic marker	^94^
Gallbladder cancer	circFOXP1	Up	miR-370	PKLR	Regulate cell Warburg effect, promote cell proliferation, migration, invasion and inhibit cell apoptosis	Prognostic marker and therapeutic target	^73^
Pancreatic cancer	circHIPK3	Up	miR-330-5p	RASSF1	Regulate cell proliferation, invasion, migration, EMT, and apoptosis	Therapeutic target	^63^
	circFOXK2	Up	miR-942	NUF2, PDXK	Promote cell growth, migration and invasion and regulate cell cycle progression and apoptosis	Therapeutic target	^50^
	circZMYM2	Up	miR-335-5p	JMJD2C	Regulate cell proliferation and apoptosis	Therapeutic target	^64^
	circ_0006215	Up	miR-378a-3p	SERPINA	Regulate cell apoptosis and migration, Promote the occurrence and development of PC	Therapeutic target	^65^
	hsa_circ_001653	Up	miR-377	miR-377/HOXC6 axis	Regulate cell viability, cell-cycle, cell angiogenesis, invasion and apoptosis	Therapeutic target	^56^
	circ-PDE8A	Up	miR-338	MACC1/MET	Promote cell invasive growth	Diagnostic and prognostic biomarker	^49^
	circ_0000977	Up	miR-153	HI1FA, ADAM10	Immune escape from NK cells	Immune sensitizers in cancer treatment and/or prevention	^80^
	circ-LDLRAD3	Up	/	/	Correlate with lymphatic invasion, venous invasion, and metastasis	Diagnostic biomarker	^106^
Colorectal cancer	circHIPK3	Up	miR-637	Bcl-2/Beclin-1	Regulate autophagy	Prognostic marker	^102^
	hsa_circ_0079662	Up	hsa-mir-324-5p	HOXA9, TNF-α pathway	Regulate cell growth, migration and invasion, Induce oxaliplatin resistance	Therapeutic target	^103^
	circLONP2	Up	miR-17	DDX1	Promote cell aggressiveness and metastasis	Prognostic marker and therapeutic target	^90^
	circDENND4C	Up	miR-760	GLUT1	Promote cell proliferation, migration and glycolysis	Therapeutic target	^75^
	circITGA7	Down	miR-370-3p	ITGA7	Inhibit cell growth and metastasis	Therapeutic target	^42^
	hsa_circ_0007142	Up	miR-103a-2-5p	/	Promote cell proliferation, migration, and invasion	Therapeutic target	^53^
	hsa_circ_0007534	Up	/	/	Correlate with clinical classifications, metastatic phenotype, poor differentiation and poor prognosis	Diagnostic and prognostic biomarker	^128^
	circ_0009361	Down	miR-582	APC2 and Wnt/β-catenin pathway	Promote cell proliferation, epithelial-mesenchymal transition (EMT), migration, and invasion	Therapeutic target	^91^
	circVAPA	Up	miR-125a	CREB5	Regulate cell cycle progression, cell migration, invasion and glycolysis	Therapeutic target	^74^
	circ_0136666	Up	miR-136	SH2B1	Regulate cell proliferation, migration, invasion and cell cycle	Prognostic marker	^51^
	circRNA_103809	Down	miR-532-3p	FOXO4	Promote cell proliferation and migration	Therapeutic target	^127^
	circ_001569	Up	miR-145	E2F5, BAG4, FMNL2	Promote cell proliferation and invasion	Therapeutic target	^92^

### Cell proliferation, differentiation, and cell cycle regulation

Excessive proliferation, impaired differentiation and maturation, and an imbalance in cell cycle regulation are features of cancer cells and important mechanisms of cancer cell hyperproliferation^47^. CircRNAs play crucial roles in the proliferation, differentiation, and cell cycle regulation of gastrointestinal cancer cells (**Figure 3A**). CircRNAs affect cell proliferation and the cell cycle mainly by regulating downstream miRNAs and directly or indirectly participating in signal transduction pathways or interactions with proteins. For example, in GC, overexpression of ciRS-7 blocked the inhibitory effect of miR-7 by antagonizing the miR-7-mediated PTEN/PI3K/AKT signaling pathway, which ultimately promoted the proliferation of cancer cells^48^. In pancreatic cancer, circ-PDE8A was demonstrated to stimulate cell growth by functioning as a competitive endogenous RNA (ceRNA) to absorb miR-338 and regulate the miR-338/MACC1/MET pathway, whereas circFOXK2 was found to be involved in the cell cycle and proliferation by interacting with RBPs and sponging miR-942 to promote cancer progression^49,50^. Similarly, in CRC, circ_0136666 increases SH2B1 levels by competitively binding miR-136 to participate in the cell cycle and facilitate cancer cell proliferation^51^. Moreover, some circRNAs, such as circSMARCA5 and circ_100395 and hsa_circ_0007142, regulate cell proliferation and cell cycle regulation, and also affect cell differentiation, which contributes to the cancer burden and poor prognoses of patients^37,52,53^. Consistent with these observations, many other circRNAs, such as circ_0008035 sponging miR-599 and circPDSS1 sponging miR-186-5p in GC, circ_101280 sponging miR-375, circSLC3A2 sponging miR-490-3p and circABCB10 sponging miR-670-3p in HCC, hsa_circ_001653 sponging miR-377 in pancreatic cancer, and hsa_circ_0006168 sponging miR-384 in esophageal cancer have been shown to affect cell proliferation and cell cycle regulation and differentiation by regulating cancer-related signaling pathways or interactions with proteins^54–60^. Overall, circRNAs regulate cell growth and differentiation and directly affect patient clinical characteristics, such as cancer diameter, and cancer growth rate and differentiation during cancer progression. Targeting specific circRNAs that are associated with cell proliferation, differentiation, or the cell cycle is therefore a potential novel approach to inhibit cancer growth.

### Cell apoptosis regulation

The growth rate of cancer is mainly determined by the ratio of cell proliferation to cell death, and inhibition of apoptosis is another important factor for the excessive growth of cancer cells^47^. CircRNAs participate in the regulation of cell apoptosis (**Figure 3B**). Likewise, “miRNA sponging” is considered the main mechanism by which circRNAs regulate cell apoptosis. For example, circ-NOTCH1 has been shown to reduce GC cell apoptosis by sponging miR-637 and then upregulating the expression of its target gene, *Apelin*^61^. Similarly, circ_0000267 has been shown to sponge miR-646 to attenuate cell apoptosis in HCC cells^62^. Consistent with this observation, circHIPK3, circZMYM2, circ_0006215, circAKT3, circ_0101432, and hsa_circ_0000523 also act as “miRNA sponges” to regulate the apoptosis of various cancer cells, including pancreatic cancer, CRC, HCC, and GC cells^63–68^. Importantly, some circRNAs are also involved in regulating the expression of apoptosis-related proteins and/or related signaling pathways. For example, circ-IGF1R activates a variety of apoptotic effectors, including the apoptotic inhibitors B-cell lymphoma-2 (Bcl-2) and Bcl-2-associated X protein (Bax), thereby exerting anti-apoptotic effects by activating the PI3K/AKT signaling pathway, subsequently influencing HCC cell proliferation, apoptosis, and cell cycle progression^69^. Similarly, circZNF609 has been shown to induce cell apoptosis by activating the expression of the *Bax* and *p53* genes and reducing Bcl-2 expression in CRC cells^70^. Thus, circRNAs are important players in apoptosis regulation. The development of apoptosis-inducing targets might therefore provide new perspectives for cancer research.

### Cell metabolism regulation

Cancer cell energy metabolism is mainly mediated through the glycolytic pathway, that is, the Warburg effect, which enables cancer cells to maintain high intensity metabolic activities even in hypoxic environments^71^. CircRNAs have been shown to participate in the regulation of cellular metabolism-related factors by functioning as miRNA sponges or being involved in signaling pathways. For example, hsa_circ_0006168 regulates glycolysis in esophageal cancer cells by competitively binding miR-384 to enhance the expression of retinoblastoma-binding protein 7 (RBBP7), subsequently activating the S6K/S6 pathway^58^. In addition, circRNAs were also found to be involved in glycolysis or glutamine catabolism by regulating transporters, enzymes, or transcription regulators^72^. In gallbladder cancer, circFOXP1 stimulated the cell Warburg effect by interacting with the RBP polypyrimidine Tract-binding protein 1 (PTBP1), which significantly increased the expression of pyruvate kinase PKLR and protected PKLR mRNA from degradation^73^. Likewise, in CRC, circDENND4C promoted cancer cell glycolysis through the increased expression of glucose transporter type 1 (GLUT1) by sponging miR-760, whereas circVAPA participated in glycolysis *via* its effect on cAMP response element-binding 5 (CREB5) by sponging miR-125a^74,75^. Moreover, a recent study revealed that circHECTD1 facilitated glutaminolysis in GC progression by inhibiting miR-1256 and activating the β-catenin/c-Myc signaling pathway^76^. Together, these studies indicate that circRNAs are closely related to the regulation of gastrointestinal cancer cell metabolism (**Figure 3C**).

### Immune escape

Through immune surveillance mechanisms, immune cells can recognize and eliminate cancer or cancer-transformed cells, exerting anticancer effects. Cancer cells can escape immune surveillance by reducing antigen expression^47^. Preliminary evidence has shown that some circRNAs, such as circUBAP2 and circTRIM33-12, participate in cancer immune escape by directly or indirectly reducing the expression of surface antigens. In pancreatic cancer, circUBAP2 regulates the expression of chemokine receptor 4 (CXCR4) and zinc finger E-box-binding homeobox1 (ZEB1) and inhibits the antigen presentation of cancer cells (**Figure 3D**), leading to tumor infiltration by immune cells and immune escape through a competitive endogenous circRNA network^77^. Similarly, circTRIM33-12 was also found to affect immune evasion of cancer cells by sponging miR-191, subsequently affecting the expression of tet methylcytosine dioxygenases 1 (TET1), which is the target gene of miR-191 in HCC^78^. The abnormal expression of both circUBAP2 and circTRIM33-12 ultimately leads to immune escape of cancer cells, which is associated with poor clinical outcomes. However, other circRNAs participate in cancer immune escape *via* dysfunctional activation of natural killer (NK) cells. For instance, in HCC, circARSP91 was shown to upregulate the expression of UL16-binding protein-1 (ULBP1), a killer cell lectin, to increase the susceptibility of cancer cells to NK cell cytotoxicity, thus enhancing the cytotoxicity of NK cells and promoting immune surveillance^79^. In pancreatic cancer, circ_0000977 was revealed to regulate the miR-153 downstream targets of hypoxia-inducible factor 1-alpha (HIF1A), a disintegrin, and Metalloproteinase Domain 10 to modulate HIF1A-mediated immune escape of pancreatic cancer cells from NK cells^80^. Moreover, the latest evidence also shows that some circRNAs, such as hsa_circ_0020397, can enhance the expression of programmed death-ligand 1 (PD-L1), which is closely related to cancer escape from immune control^81^. Notably, the escape of cancer cells from immune surveillance often contributes to excessive cancer cell growth and metastasis, leading to adverse clinical prognoses of patients^82^. Although limited circRNAs correlated with immune escape have been found, there is considerable evidence that circRNAs play important roles in cancer immune escape. More immune escape-related circRNAs need to be discovered in the future, and the specific mechanisms require further in-depth studies.

### Invasion and metastasis regulation

Malignancies can grow abnormally at the primary site and adjacent tissues, and can also spread to distant organs. Some dysregulated circRNAs were discovered to exert regulatory effects on the invasion and metastasis of malignancies (**Figure 3E**), which seriously affected the clinicopathological features and prognoses of cancer patients. The “miRNA sponge” function is their main mechanism. In colon polyps and colon cancer tissues, a high level of circCCDC66 has been found to be related to pathological processes including cell proliferation, migration, invasion, and anchorage-independent growth, which contributes to cancer development and poor prognoses of patients^83^. In pancreatic cancer, tissue ciRS-7 levels are related to several malignant tendencies, such as venous invasion and lymph node metastasis^84^. Mechanistically, upregulated ciRS-7 was found to inhibit the expression of miR-7 and upregulate the expression of epidermal growth factor receptor (EGFR) and signal transducer and activator of transcription 3 (STAT3), to affect the proliferation and invasion of pancreatic cancer cells^84^. Conversely, in esophageal cancer, increased ciRS-7 has been shown to function as a ceRNA to activate miR-7/KLF4 and NF-κB signaling, further inducing the migration and invasion of cancer cells^85^. Likewise, other circRNAs, such as circ_0000337, hsa_circ_0006168, circUBAP2, circPIP5K1A, circNRIP1, circ_0006215, circLONP2, circ_0009361, circRNA_001569, circ_0078710, and circRHOT1 have also been shown to regulate cell invasion and metastasis in gastrointestinal malignancies by acting as miRNA sponges or ceRNAs^35,65,86–94^.

CircRNAs also participate in the regulation of invasion and metastasis by recruiting protein factors or promoting the EMT. Sun et al.^95^ found that circ-ADD3 enhanced the interaction between cyclin-dependent kinase 1 (CDK1) and histone-lysine N-methyltransferase (EZH2), resulting in ubiquitination and degradation of EZH2 through phosphorylation at Thr-345 and Thr-487 during the progression of HCC. The subsequent decrease in EZH2 levels significantly increased the levels of a number of anti-metastatic genes, including *circ-ADD3*, by reducing the level of H3K27me3 on their promoter regions, thus forming a regulatory circuit to inhibit HCC metastasis^95^. Chen et al.^52^ demonstrated that circ_100395 participated in the EMT process to regulate HCC cell metastasis. Mechanistically, downregulated circ_100395 suppresses the EMT by directly binding to the downstream factor miR-1228, while EMT pathway dysfunction involves the antimetastatic effect in HCC cells^52^. In addition, the roles of other circRNAs, such as hsa_circ_0012563 in esophageal cancer, circRNA_0023642 in GC, and circFNDC3B and circRNA_101951 in CRC, in invasion and metastasis by regulating EMT pathways, have been reported^96–99^. Together, these findings provide new evidence that circRNAs are directly involved in the invasion and metastasis of gastrointestinal malignancies, which might be responsible for the degree of cell malignancy, distant metastasis, postoperative recurrence, and even the poor clinical outcomes of patients.

### Angiogenesis regulation

Neovascularization plays a key role in cancer growth, providing necessary nutrients for cell growth and tissue metabolism. Angiogenesis is an important factor for continuous tumor growth and metastasis^47^. A growing number of circRNAs have been discovered that regulated angiogenesis and increased the malignant degree of gastrointestinal cancers (**Figure 3F**). For example, circRNA-100338 levels were greatly increased in both highly metastatic HCC cells and their secreted exosomes, and enhanced the proliferation, invasive abilities, angiogenesis, permeability, and the formation of vasculogenic mimicry (VM) of human umbilical vein endothelial cells^100^. Circ_0000092 was also increased in HCC tissues and cell lines and promoted the progression and angiogenesis of HCC by regulating the miR-338-3p/HN1 axis^101^. Moreover, in pancreatic cancer, hsa_circ_001653 was demonstrated to regulate cancer angiogenesis by acting as a miR-377 sponge and activating the miR-377/HOXC6 axis^56^. Thus, circRNAs are also involved in the regulation of cancer angiogenesis by acting as miRNA sponges. The development of circRNA-targeted antiangiogenic drugs is a potential therapeutic strategy for cancer, but it is mainly dependent on the identification of more vascular proliferative RNAs and the characterization of their molecular mechanisms.

### Drug resistance regulation

Comprehensive treatment based on surgery and chemotherapy is currently the main treatment method for gastrointestinal malignancies. Recently, some circRNAs related to chemotherapeutic drug resistance have been identified (**Figure 3G**). Dysregulated circRNAs in gastrointestinal cancer tissues have important impacts on the efficacy of clinical chemotherapy in patients, often leading to treatment failure or cancer recurrence and metastasis. For example, in recurrent CRC and oxaliplatin (OXA) resistant patients, the upregulated circHIPK3 in tissues promotes oxaliplatin resistance and cancer progression by sponging miR-637, increasing the expression of STAT3, which subsequently activates the downstream Bcl-2/beclin1 signaling pathway^102^. Importantly, in patients with postoperative OXA-based adjuvant chemotherapy, the circHIPK3 level was found to be negatively correlated with 5-year disease-free survival and overall survival rates, suggesting that circHIPK3 has important impacts on the efficacy of clinical OXA chemotherapy in patients^102^. In another study, the upregulation of hsa_circ_0079662 was demonstrated to promote CRC migration and invasion and induce oxaliplatin resistance *via* the TNF-α pathway^103^. Similarly, in GC patients, circAKT3 was significantly overexpressed in cisplatin-resistant GC tissues and cells. The dysregulated circAKT3 acts as a miR-198 sponge to activate the PI3K/AKT signaling pathway and upregulate the DNA damage repair molecule breast cancer 1 (BRCA1), which inhibits cancer cell apoptosis and also leads to resistance in DNA-damaging CDDP-based chemotherapy^66^. Likewise, the decreased circRNA_101505 can function as a ceRNA by sponging miR-103 to target and upregulate the expression of the downstream oxidored-nitro domain-containing protein 1, which confers cisplatin resistance to cancer cells, resulting in poor overall survival of HCC patients^104^. This evidence suggests that targeting drug resistance-related circRNAs may increase the sensitivity of patients to chemotherapeutic drugs, and reduce or reverse drug resistance and improve the prognoses.

## Potential clinical applications of circRNAs in gastrointestinal malignancies

CircRNAs are characterized by their abundance, high stability, extensive functions, and certain tissue, time, and disease specificities, suggesting their potential clinical applications in the diagnoses and treatments of gastrointestinal tumors.

### Biomarkers for gastrointestinal malignancies

#### Plasma circRNAs

In cancer screening, plasma biomarkers are particularly important because of their unique advantages, such as convenience, repeatable sampling, and minimal trauma. Unfortunately, the current biomarkers used in the clinic have suboptimal sensitivity and specificity, which limits further clinical application, especially for early screening.

Recent studies have shown that some circulating plasma circRNAs have better diagnostic potentials than traditional markers such as CEA, CA19-9, and AFP, and the combined use of these markers significantly improves the diagnostic efficacy. Plasma hsa_circ_0003998 levels in HCC were significantly higher than those in hepatitis B patients and healthy individuals, and decreased rapidly after surgery^105^. A study of the ability of hsa_circ_0003998 as a plasma marker to distinguish HCC patients from hepatitis B patients and healthy individuals reported that the sensitivity and specificity of plasma hsa_circ_0003998 were 0.83 and 0.7 and 0.8 and 0.84, respectively^105^. Moreover, the combined use of hsa_circ_0003998 and AFP further improved the diagnosis efficacy with the highest sensitivity and specificity of 0.88 and 0.92, respectively^105^. In pancreatic cancer, circ-LDLRAD3 is an ideal marker of diagnosis and invasion capacity. The sensitivity of plasma circ-LDLRAD3 combined with CA19-9 in the diagnosis of pancreatic cancer was 80.33%, and the specificity was up to 93.55%, which was significantly higher than that of CA19-9 or circ-LDLRAD3 alone^106^. Similarly, in early GC identification, plasma hsa_circ_0006848 combined with CEA, CA19-9, and CA72-4 also improved the diagnostic value, with a sensitivity and specificity of 73.3% and 90.0%, respectively^107^.

Although combined detection significantly improves efficiency, single plasma circRNA markers still have excellent performance. When used alone, circSMARCA5 has the potential to be an ideal biomarker for HCC screening, especially in patients with low AFP levels. Plasma circSMARCA5 has unique diagnostic value in HCC with a sensitivity and specificity of 86.67% and 89.32%, respectively, and also displays good predictive value for distinguishing HCC from hepatitis or cirrhosis patients with AFP levels below 200 ng/mL^108^. In esophageal cancer screening, the sensitivity and specificity of plasma hsa_circ_0001946 were up to 92% and 80%, respectively, and those of hsa_circ_0043603 were 64% and 92%, respectively, which were both significantly higher than those of CEA^109^. In GC screening, the sensitivity of plasma hsa_circ_0000181 reached 99.0% when the cutoff value was 7.27^110^. Likewise, more circRNAs, such as hsa_circ_0000976, hsa_circ_0007750, and hsa_circ_0139897 in HCC and circ-ABCC1, circ-CCDC66, and circ-ABCC1 in CRC, have good clinical diagnostic values, whether in combination or used alone^111,112^. Although an increasing number of circRNAs in cancer tissues have been identified, the diagnostic value of most circRNAs is still largely unknown and needs further study.

#### Exosomal circRNAs

Exosomes play important roles in various pathophysiological processes such as intercellular signal transmission^113^. Exosomal circRNAs are involved in multiple pathological processes, including the EMT, tumor cell proliferation, and tumor angiogenesis. In addition, exosome circRNAs are considered indicators for the early diagnosis and prognostic evaluation of multiple cancers^114^. For example, circHIPK3, was significantly upregulated in a cholangiocarcinoma (CCA) cell line, tissues, and plasma exosomes, and was found to promote the proliferation, invasion, and migration of CCA cells^115^. Moreover, exosome-mediated circHIPK3 had a cancer-promoting effect on adjacent normal cells^116^. Thus, circHIPK3, which is enriched and stably expressed in exosomes, was considered a noninvasive biomarker for CCA diagnosis. Dysregulated circ-PDE8A in pancreatic cancer was shown to be associated with cancer invasion, progression, and low survival times^49^. Tumor cell-excreted circ-PDE8A could enter the peripheral circulation through exosomes and was easy to detect^49^. Therefore, circ-PDE8A is predicted to be a diagnostic indicator for cancer invasion evaluation and prognosis of pancreatic cancer. In GC, plasma exosomal hsa_circ_0065149 is thought to be an important indicator for early screening. The sensitivity and specificity of exosomal hsa_circ_0065149 were 48.7% and 90.2%, respectively^116^. Regarding sensitivity, exosomal hsa_circ_0065149 sensitivity was significantly higher than that of traditional biomarkers such as CEA (4.3%), CA19-9 (4.8%), and CA-125 (1.9%)^116^.

The study of exosomal circRNAs as tumor indicators of the digestive system is still in its infancy, and only a few circRNAs have been discovered. Moreover, research on exosomal circRNAs as biomarkers still faces many challenges, of which the low concentration of exosomes in body fluids, low abundance of circRNAs in exosomes, relatively complicated detection processes of exosomes in fluids, and the need for a larger fluid sample have greatly restricted the in-depth study of exosomal circRNAs. Future improvements in the above 4 aspects are expected to help further reveal the value of exosomal circRNAs as biomarkers.

#### Gastric juice circRNAs

Gastric juice, a gastric endocrine product secreted by gastric mucosal epithelial cells, fundus glandular cells, and cervical mucous cells, has an obvious advantage for its use in GC due to its organ specificity. Some circRNAs, such as hsa_circ_0014717 and hsa_circ_0065149, were successfully extracted and detected in gastric juice by RT-PCR and DNA sequencing^116,117^. A preliminary study showed that hsa_circ_0014717 in gastric juice was stable enough to meet the needs of clinical diagnoses, and the change in its levels could indicate some specific pathological changes in gastric mucosa^117^. At present, research on circRNAs in gastric juice is still in its infancy, and most related clinically significant and functional mechanisms are unclear. However, the stability of gastric juice circRNAs indicates that they may have great potential in the future.

### Prognostic indicators of gastrointestinal malignancies

Some dysregulated circRNAs related to the clinicopathological characteristics, malignant biological behavior (such as tumor growth, differentiation, and metastasis), and prognosis of gastrointestinal cancers are important regulators of tumorigenesis and progression, and also provide key indicators for evaluating the prognoses of patients with malignancies. For example, CDR1as, a classical circRNA overexpressed in multiple cancers, including HCC, CRC, and CCA, was found to affect patient clinical prognoses by acting as a ceRNA to sponge miRNAs^118–120^. CDR1as levels were shown to be positively correlated with multiple clinicopathological features and malignant biological behaviors, including cancer size and tumor-node-metastasis (TNM) stage^118–120^. Furthermore, patients with higher CDR1as levels in both CRC and CCA had a worse overall survival than patients with lower CDR1as expressions^118,120^. In addition, CCA patients with elevated CDR1as levels had a higher risk of metastasis and postoperative recurrence rate^118^. Likewise, in GC, patients with lower circPVT1 levels were shown to have a worse overall survival (median survival of 20 months *vs*. 46 months) and disease-free survival (median survival of 17 months *vs*. 36 months) than those with higher circPVT1 levels^121^. In HCC, the upregulation of hsa_circ_0003998 was shown to be associated with higher serum AFP levels, larger cancer size, poorer differentiation, microvascular invasion, and lower overall survival rate, which were prognostic factors for HCC patients^105^. Similarly, other dysregulated circRNAs associated with poor clinical outcome of malignancies, such as circ_100876 and hsa_circ_0004771 in esophageal cancer, circLARP4 and circYAP1 in GC, circMTO1 and circSETD3 in HCC and circRNA_103809 and hsa_circ_0007534 in CRC, have also been identified as prognostic indicators, providing clues for the prognostic evaluation of gastrointestinal malignancies^4,122–128^.

Notably, circRNAs related to cancer prognosis in certain tissues are also present in plasma. These fluid circRNAs can be used as cancer diagnostic markers and also as references for prognostic evaluations. For example, in pancreatic cancer, overexpression of circ-PDE8A in tissues was correlated with lymphatic invasion, TNM stage, and poor survival rate, whereas plasma exosomal circ-PDE8A levels were also closely related to cancer progression and patient overall survival, providing evidence for cancer diagnosis or progression^49^. Furthermore, hsa_circ_0007534 was found to be significantly upregulated in both CRC tissues and plasma. Importantly, hsa_circ_0007534 in CRC plasma was associated with clinical classification, metastatic phenotype, and poor differentiation, and was positively correlated with poor survival of CRC patients, providing an indicator for prognostic evaluation^128^. Likewise, other plasma circRNAs, such as hsa_circ_0000419 and hsa_circ_0065149 in GC, circ-LDLRAD3 and circ-PDE8A in pancreatic cancer, and hsa_circ_0001946 and circ-SLC7A5 in esophageal cancer, have also been associated with clinicopathological features, malignant biological behaviors, and prognoses of cancers, and were shown to serve as prognostic indicators^5,49,106,109,116,129^.

Overall, circRNAs are widely involved in the malignancy of tumors, and circRNAs in tissues and body fluids are considered independent biomarkers, providing clues for the prognostic evaluation of gastrointestinal malignancies. Specific detection of these circRNAs may improve the current dilemma of prognostic evaluations in patients.

### Therapeutic targets of gastrointestinal malignancies

Specific targeted upregulation or silencing of dysregulated circRNA expression can suppress cancer cell growth, proliferation and invasion, and also improve differentiation, apoptosis, and multidrug resistance. For this reason, circRNAs represent potential targets for the treatment of gastrointestinal malignancies, which may largely reverse the difficulties in future cancer treatments. For example, circHIPK3, a well-known multifunctional circRNA, acts as an oncogene that is significantly overexpressed in esophageal cancer, GC, CRC, and pancreatic cancer, and widely promotes the occurrence and development of multiple cancers by acting as a “miRNA sponge” or modulating cancer-related signaling pathways^63,102,130–133^. Patients with high levels of circHIPK3 have been found to be associated with worse clinicopathological features and prognoses, such as larger tumor size, worse cell differentiation, and higher risk of recurrence and metastasis, while silencing of circHIPK3 inhibited cancer cell proliferation, migration, and invasion, induced apoptosis, and even sensitized cells to oxaliplatin and gemcitabine in these malignancies^63,102,130–133^. Similarly, in HCC, reversing the overexpression of circFBXO11 in cells significantly repressed carcinoma progression and oxaliplatin resistance by sponging miR-605, subsequently affecting the expression of FOXO3 and ABCB1, which are target genes of miR-605, suggesting that circFBXO11 has the potential to improve patient prognosis and survival^134^. Consistent with these observations, other low-expressed circRNAs act as tumor suppressor genes, such as circMTO1 and circHIAT1 in HCC, circITGA7 and hsa_circ_0000523 in CRC, circPSMC3 and circ_0027599 in GC, hsa_circ_0001649 and hsa_circ_001653 in pancreatic cancer, circ-Foxo3 and cir-ITCH in esophageal cancer, and circMAN2B2 and hsa_circ_0056836 in HCC also showed similar characteristics when their expression levels were restored^41,42,56,67,126,135–142^. Overall, growing evidence indicates that circRNAs provide potential therapeutic targets for the treatment of gastrointestinal malignancies. Therefore, future studies aimed at tumor-specific circRNAs and designing and implementing the transfection of vectors with precise targeted overexpression or silencing of circRNAs will provide a new scheme in cancer treatment.

## Perspectives

Over the past several years, much progress has been made in understanding the biological functions of circRNAs and the mechanisms responsible for their contributions to carcinogenesis of the digestive system. These investigations have shown that circRNAs play key roles in the development and progression of gastrointestinal malignancies, and are associated with patient prognoses and clinical outcomes^143,144^. Research in this field therefore holds much promise in identifying novel diagnostic biomarkers and new treatment targets for gastrointestinal malignancies.

However, several outstanding limitations and challenges remain. First, investigation of more dysregulated circRNAs in carcinogenesis is still in its infancy, and the mechanisms responsible for their contributions to cancer biology still remain to be identified in gastrointestinal malignancies. In the future, more dysregulated circRNAs need to be identified by developing high-throughput RNA sequencing technology. Second, there are no uniform naming rules for circRNAs to date, which will certainly increase some unnecessary contradictions and problems in research work. Therefore, considering the large number of circRNAs, it is urgent to establish a uniform naming convention to unify the data. Third, although there are many circRNA databases available to meet different research needs, they still lack relevance. In addition, these databases generally lack clinical data of specific circRNA-related diseases, and the data need to be constantly updated to meet new requirements. Fourth, the understanding of the roles of circRNAs in cancer is not comprehensive enough. Current studies on the function and mechanism of circRNAs in cancer are mostly limited to a single or a few circRNAs, which lack systematic approaches, comprehensiveness, and integration. In addition, mechanistic studies have mainly focused on downstream targets, such as miRNA sponges, binding with RBPs, regulating alternative splicing and transcription of parental genes, and translation into proteins. Other mechanisms, such as transcriptional regulation, and transport and degradation of circRNAs, are poorly understood and need to be characterized in the future. Fifth, current clinical transformation studies of circRNAs are insufficient. Most circRNA studies are still theoretical. The clinical significance and potential applications in gastrointestinal malignancies are still poorly understood. More attention should be paid to clinical transformation.

In conclusion, recent studies have confirmed that circRNAs influence the occurrence and evolution of gastrointestinal tumors through various regulatory mechanisms. To date, circRNAs are expected to be ideal biomarkers for clinical screening, prognostic evaluation and gene therapy targets of gastrointestinal malignancies. Because tumorigenesis and development are complex processes involving multiple factors and multiple stages, the molecular mechanisms of circRNAs in gastrointestinal malignancies need further study.

## References

[1] Ferlay J, Colombet M, Soerjomataram I, Mathers C, Parkin DM, Piñeros M (2019). Estimating the global cancer incidence and mortality in 2018: GLOBOCAN sources and methods. Int J Cancer.

[2] Lu R, Shao Y, Tao X, Ye G, Xiao B, Guo J (2019). Clinical significances of hsa_circ_0067582 and hsa_circ_0005758 in gastric cancer tissues. J Clin Lab Anal.

[3] Ruan H, Deng X, Dong L, Yang D, Xu Y, Peng H (2019). Circular RNA circ_0002138 is down-regulated and suppresses cell proliferation in colorectal cancer. Biomed Pharmacother.

[4] Xu L, Feng X, Hao X, Wang P, Zhang Y, Zheng X (2019). CircSETD3 (Hsa_circ_0000567) acts as a sponge for microRNA-421 inhibiting hepatocellular carcinoma growth. J Exp Clin Cancer Res.

[5] Tao X, Shao Y, Lu R, Ye Q, Xiao B, Ye G (2020). Clinical significance of hsa_circ_0000419 in gastric cancer screening and prognosis estimation. Pathol Res Pract.

[6] Ruan Y, Li Z, Shen Y, Li T, Zhang H, Guo J (2020). Functions of circular RNAs and their potential applications in gastric cancer. Expert Rev Gastroenterol Hepatol.

[7] Luo B, Tang CM, Chen JS (2019). circRNA and gastrointestinal cancer. J Cell Biochem.

[8] Shang Q, Yang Z, Jia R, Ge S (2019). The novel roles of circRNAs in human cancer. Mol Cancer.

[9] Wu J, Qi X, Liu L, Hu X, Liu J, Yang J (2019). Emerging epigenetic regulation of circular RNAs in human cancer. Mol Ther Nucleic Acids.

[10] Chen Y, Yang F, Fang E, Xiao W, Mei H, Li H (2019). Circular RNA circAGO2 drives cancer progression through facilitating HuR-repressed functions of AGO2-miRNA complexes. Cell Death Differ.

[11] Zhang Z, Xie Q, He D, Ling Y, Li Y, Li J (2018). Circular RNA: new star, new hope in cancer. BMC Cancer.

[12] Wu Q, Li P, Wu M, Liu Q (2019). Deregulation of circular RNAs in cancer from the perspectives of aberrant biogenesis, transport and removal. Front Genet.

[13] Kelly S, Greenman C, Cook PR, Papantonis A (2015). Exon skipping is correlated with exon circularization. J Mol Biol.

[14] Ivanov A, Memczak S, Wyler E, Torti F, Porath HT, Orejuela MR (2015). Analysis of intron sequences reveals hallmarks of circular RNA biogenesis in animals. Cell Rep.

[15] Panda AC, De S, Grammatikakis I, Munk R, Yang X, Piao Y (2017). High-purity circular RNA isolation method (RPAD) reveals vast collection of intronic circRNAs. Nucleic Acids Res.

[16] Li Z, Huang C, Bao C, Chen L, Lin M, Wang X (2015). Exon-intron circular RNAs regulate transcription in the nucleus. Nat Struct Mol Biol.

[17] Conn SJ, Pillman KA, Toubia J, Conn VM, Salmanidis M, Phillips CA (2015). The RNA binding protein quaking regulates formation of circRNAs. Cell.

[18] Lu Z, Filonov GS, Noto JJ, Schmidt CA, Hatkevich TL, Wen Y (2015). Metazoan tRNA introns generate stable circular RNAs in vivo. RNA.

[19] Noto JJ, Schmidt CA, Matera AG (2017). Engineering and expressing circular RNAs via tRNA splicing. RNA Biol.

[20] Wu K, Liao X, Gong Y, He J, Zhou JK, Tan S (2019). Circular RNA F-circSR derived from SLC34A2-ROS1 fusion gene promotes cell migration in non-small cell lung cancer. Mol Cancer.

[21] Guarnerio J, Bezzi M, Jeong JC, Paffenholz SV, Berry K, Naldini MM (2016). Oncogenic role of fusion-circRNAs derived from cancer-associated chromosomal translocations. Cell.

[22] Toptan T, Abere B, Nalesnik MA, Swerdlow SH, Ranganathan S, Lee N (2018). Circular DNA tumor viruses make circular RNAs. Proc Natl Acad Sci U S A.

[23] Liu X, Wang X, Li J, Hu S, Deng Y, Yin H (2020). Identification of mecciRNAs and their roles in the mitochondrial entry of proteins. Sci China Life Sci.

[24] Jeck WR, Sorrentino JA, Wang K, Slevin MK, Burd CE, Liu J (2013). Circular RNAs are abundant, conserved, and associated with ALU repeats. RNA.

[25] Zhang XO, Wang HB, Zhang Y, Lu X, Chen LL, Yang L (2014). Complementary sequence-mediated exon circularization. Cell.

[26] Schwanhäusser B, Busse D, Li N, Dittmar G, Schuchhardt J, Wolf J (2011). Global quantification of mammalian gene expression control. Nature.

[27] Chandola U, Das R, Panda B (2015). Role of the N6-methyladenosine RNA mark in gene regulation and its implications on development and disease. Brief Funct Genomics.

[28] Park OH, Ha H, Lee Y, Boo SH, Kwon DH, Song HK (2019). Endoribonucleolytic cleavage of m(6)A-containing RNAs by RNase P/MRP complex. Mol Cell.

[29] Hammond SM, Boettcher S, Caudy AA, Kobayashi R, Hannon GJ (2001). Argonaute2, a link between genetic and biochemical analyses of RNAi. Science.

[30] Kleaveland B, Shi CY, Stefano J, Bartel DP (2018). A network of noncoding regulatory RNAs acts in the mammalian brain. Cell.

[31] Hansen TB, Wiklund ED, Bramsen JB, Villadsen SB, Statham AL, Clark SJ (2011). miRNA-dependent gene silencing involving Ago2-mediated cleavage of a circular antisense RNA. Embo J.

[32] Lasda E, Parker R (2016). Circular RNAs co-precipitate with extracellular vesicles: a possible mechanism for circRNA clearance. PLoS One.

[33] Hansen TB, Jensen TI, Clausen BH, Bramsen JB, Finsen B, Damgaard CK (2013). Natural RNA circles function as efficient microRNA sponges. Nature.

[34] Hansen TB, Kjems J, Damgaard CK (2013). Circular RNA and miR-7 in cancer. Cancer Res.

[35] Zhang X, Wang S, Wang H, Cao J, Huang X, Chen Z (2019). Circular RNA circNRIP1 acts as a microRNA-149-5p sponge to promote gastric cancer progression via the AKT1/mTOR pathway. Mol Cancer.

[36] Szabo L, Salzman J (2016). Detecting circular RNAs: bioinformatic and experimental challenges. Nat Rev Genet.

[37] Barbagallo D, Caponnetto A, Brex D, Mirabella F, Barbagallo C, Lauretta G (2019). CircSMARCA5 regulates VEGFA mRNA splicing and angiogenesis in glioblastoma multiforme through the binding of SRSF1. Cancers (Basel).

[38] Yang F, Fang E, Mei H, Chen Y, Li H, Li D (2019). Cis-acting circ-CTNNB1 promotes β-catenin signaling and cancer progression via DDX3-mediated transactivation of YY1. Cancer Res.

[39] Qin M, Wei G, Sun X (2018). Circ-UBR5: an exonic circular RNA and novel small nuclear RNA involved in RNA splicing. Biochem Biophys Res Commun.

[40] Ashwal-Fluss R, Meyer M, Pamudurti NR, Ivanov A, Bartok O, Hanan M (2014). circRNA biogenesis competes with pre-mRNA splicing. Mol Cell.

[41] Li F, Zhang L, Li W, Deng J, Zheng J, An M (2015). Circular RNA ITCH has inhibitory effect on ESCC by suppressing the Wnt/β-catenin pathway. Oncotarget.

[42] Li X, Wang J, Zhang C, Lin C, Zhang J, Zhang W (2018). Circular RNA circITGA7 inhibits colorectal cancer growth and metastasis by modulating the Ras pathway and upregulating transcription of its host gene ITGA7. J Pathol.

[43] Wang Y, Wang Z (2015). Efficient backsplicing produces translatable circular mRNAs. RNA.

[44] Legnini I, Di Timoteo G, Rossi F, Morlando M, Briganti F, Sthandier O (2017). Circ-ZNF609 is a circular RNA that can be translated and functions in myogenesis. Mol Cell.

[45] van Heesch S, Witte F, Schneider-Lunitz V, Schulz JF, Adami E, Faber AB (2019). The translational landscape of the human heart. Cell.

[46] Yang Y, Fan X, Mao M, Song X, Wu P, Zhang Y (2017). Extensive translation of circular RNAs driven by N(6)-methyladenosine. Cell Res.

[47] Hanahan D, Weinberg RA (2011). Hallmarks of cancer: the next generation. Cell.

[48] Pan H, Li T, Jiang Y, Pan C, Ding Y, Huang Z (2018). Overexpression of circular RNA ciRS-7 abrogates the tumor suppressive effect of miR-7 on gastric cancer via PTEN/PI3K/AKT signaling pathway. J Cell Biochem.

[49] Li Z, Yanfang W, Li J, Jiang P, Peng T, Chen K (2018). Tumor-released exosomal circular RNA PDE8A promotes invasive growth via the miR-338/MACC1/MET pathway in pancreatic cancer. Cancer Lett.

[50] Wong CH, Lou UK, Li Y, Chan SL, Tong JHM, To KF (2020). CircFOXK2 promotes growth and metastasis of pancreatic ductal adenocarcinoma by complexing with RNA binding proteins and sponging MiR-942. Cancer Res.

[51] Jin C, Wang A, Liu L, Wang G, Li G (2019). Hsa_circ_0136666 promotes the proliferation and invasion of colorectal cancer through miR-136/SH2B1 axis. J Cell Physiol.

[52] Chen Q, Chen Z, Cao S, Guo B, Chen Y, Feng Z (2019). Role of CircRNAs_100395 in proliferation and metastases of liver cancer. Med Sci Monit.

[53] Zhu CL, Sha X, Wang Y, Li J, Zhang MY, Guo ZY (2019). Circular RNA hsa_circ_0007142 is upregulated and targets miR-103a-2-5p in colorectal cancer. J Oncol.

[54] Li C, Tian Y, Liang Y, Li Q (2020). Circ_0008035 contributes to cell proliferation and inhibits apoptosis and ferroptosis in gastric cancer via miR-599/EIF4A1 axis. Cancer Cell Int.

[55] Cao S, Wang G, Wang J, Li C, Zhang L (2019). Hsa_circ_101280 promotes hepatocellular carcinoma by regulating miR-375/JAK2. Immunol Cell Biol.

[56] Shi H, Li H, Zhen T, Dong Y, Pei X, Zhang X (2020). hsa_circ_001653 implicates in the development of pancreatic ductal adenocarcinoma by regulating microRNA-377-mediated HOXC6 axis. Mol Ther Nucleic Acids.

[57] Ouyang Y, Li Y, Huang Y, Li X, Zhu Y, Long Y (2019). CircRNA circPDSS1 promotes the gastric cancer progression by sponging miR-186-5p and modulating NEK2. J Cell Physiol.

[58] Xie ZF, Li HT, Xie SH, Ma M (2020). Circular RNA hsa_circ_0006168 contributes to cell proliferation, migration and invasion in esophageal cancer by regulating miR-384/RBBP7 axis via activation of S6K/S6 pathway. Eur Rev Med Pharmacol Sci.

[59] Fu Y, Cai L, Lei X, Wang D (2019). Circular RNA ABCB10 promotes hepatocellular carcinoma progression by increasing HMG20A expression by sponging miR-670-3p. Cancer Cell Int.

[60] Wang H, Chen W, Jin M, Hou L, Chen X, Zhang R (2018). CircSLC3A2 functions as an oncogenic factor in hepatocellular carcinoma by sponging miR-490-3p and regulating PPM1F expression. Mol Cancer.

[61] Guan E, Xu X, Xue F (2020). circ-NOTCH1 acts as a sponge of miR-637 and affects the expression of its target gene Apelin to regulate gastric cancer cell growth. Biochem Cell Biol.

[62] Pan H, Tang L, Jiang H, Li X, Wang R, Gao J (2019). Enhanced expression of circ_0000267 in hepatocellular carcinoma indicates poor prognosis and facilitates cell progression by sponging miR-646. J Cell Biochem.

[63] Liu Y, Xia L, Dong L, Wang J, Xiao Q, Yu X (2020). CircHIPK3 promotes gemcitabine (GEM) resistance in pancreatic cancer cells by sponging miR-330-5p and targets RASSF1. Cancer Manag Res.

[64] An Y, Cai H, Zhang Y, Liu S, Duan Y, Sun D (2018). circZMYM2 competed endogenously with miR-335-5p to regulate JMJD2C in pancreatic cancer. Cell Physiol Biochem.

[65] Zhu P, Ge N, Liu D, Yang F, Zhang K, Guo J (2018). Preliminary investigation of the function of hsa_circ_0006215 in pancreatic cancer. Oncol Lett.

[66] Huang X, Li Z, Zhang Q, Wang W, Li B, Wang L (2019). Circular RNA AKT3 upregulates PIK3R1 to enhance cisplatin resistance in gastric cancer via miR-198 suppression. Mol Cancer.

[67] Jin Y, Yu LL, Zhang B, Liu CF, Chen Y (2018). Circular RNA hsa_circ_0000523 regulates the proliferation and apoptosis of colorectal cancer cells as miRNA sponge. Braz J Med Biol Res.

[68] Zou H, Xu X, Luo L, Zhang Y, Luo L, Yao Y (2019). Hsa_circ_0101432 promotes the development of hepatocellular carcinoma (HCC) by adsorbing miR-1258 and miR-622. Cell Cycle.

[69] Fu HW, Lin X, Zhu YX, Lan X, Kuang Y, Wang YZ (2019). Circ-IGF1R has pro-proliferative and anti-apoptotic effects in HCC by activating the PI3K/AKT pathway. Gene.

[70] Zhang X, Zhao Y, Kong P, Han M, Li B (2019). Expression of circZNF609 is down-regulated in colorectal cancer tissue and promotes apoptosis in colorectal cancer cells by upregulating p53. Med Sci Monit.

[71] Warburg O (1956). On the origin of cancer cells. Science.

[72] Yu T, Wang Y, Fan Y, Fang N, Wang T, Xu T (2019). CircRNAs in cancer metabolism: a review. J Hematol Oncol.

[73] Wang S, Zhang Y, Cai Q, Ma M, Jin LY, Weng M (2019). Circular RNA FOXP1 promotes tumor progression and Warburg effect in gallbladder cancer by regulating PKLR expression. Mol Cancer.

[74] Zhang X, Xu Y, Yamaguchi K, Hu J, Zhang L, Wang J (2020). Circular RNA circVAPA knockdown suppresses colorectal cancer cell growth process by regulating miR-125a/CREB5 axis. Cancer Cell Int.

[75] Zhang ZJ, Zhang YH, Qin XJ, Wang YX, Fu J (2020). Circular RNA circDENND4C facilitates proliferation, migration and glycolysis of colorectal cancer cells through miR-760/GLUT1 axis. Eur Rev Med Pharmacol Sci.

[76] Cai J, Chen Z, Wang J, Wang J, Chen X, Liang L (2019). circHECTD1 facilitates glutaminolysis to promote gastric cancer progression by targeting miR-1256 and activating β-catenin/c-Myc signaling. Cell Death Dis.

[77] Zhao R, Ni J, Lu S, Jiang S, You L, Liu H (2019). CircUBAP2-mediated competing endogenous RNA network modulates tumorigenesis in pancreatic adenocarcinoma. Aging (Albany NY).

[78] Zhang PF, Wei CY, Huang XY, Peng R, Yang X, Lu JC (2019). Circular RNA circTRIM33-12 acts as the sponge of MicroRNA-191 to suppress hepatocellular carcinoma progression. Mol Cancer.

[79] Ma Y, Zhang C, Zhang B, Yu H, Yu Q (2019). CircRNA of AR-suppressed PABPC1 91 bp enhances the cytotoxicity of natural killer cells against hepatocellular carcinoma via upregulating UL16 binding protein 1. Oncol Lett.

[80] Ou ZL, Luo Z, Wei W, Liang S, Gao TL (2019). Hypoxia-induced shedding of MICA and HIF1A-mediated immune escape of pancreatic cancer cells from NK cells: role of circ_0000977/miR-153 axis. RNA Biol.

[81] Zhang LX, Xu LL, Wang F (2017). Hsa_circ_0020397 regulates colorectal cancer cell viability, apoptosis and invasion by promoting the expression of the miR-138 targets TERT and PD-L1. Cell Biol Int.

[82] Zhou Z, Sun B, Huang SQ, Zhao LL (2019). Roles of circular RNAs in immunoregulation and autoimmune diseases. Cell Death Dis.

[83] Hsiao KY, Lin YC, Gupta SK, Chang N, Yen L, Sun HS (2017). Noncoding effects of circular RNA CCDC66 promote colon cancer growth and metastasis. Cancer Res.

[84] Liu L, Liu FB, Huang M, Xie K, Xie QS, Liu CH (2019). Circular RNA ciRS-7 promotes the proliferation and metastasis of pancreatic cancer by regulating miR-7-mediated EGFR/STAT3 signaling pathway. Hepatobiliary Pancreat Dis Int.

[85] Huang H, Wei L, Qin T, Yang N, Li Z, Xu Z (2019). Circular RNA ciRS-7 triggers the migration and invasion of esophageal squamous cell carcinoma via miR-7/KLF4 and NF-κB signals. Cancer Biol Ther.

[86] Song H, Xu D, Shi P, He B, Li Z, Ji Y (2019). Upregulated circ RNA hsa_circ_0000337 promotes cell proliferation, migration, and invasion of esophageal squamous cell carcinoma. Cancer Manag Res.

[87] Shi Y, Guo Z, Fang N, Jiang W, Fan Y, He Y (2019). hsa_circ_0006168 sponges miR-100 and regulates mTOR to promote the proliferation, migration and invasion of esophageal squamous cell carcinoma. Biomed Pharmacother.

[88] Wu Y, Zhi L, Zhao Y, Yang L, Cai F (2020). Knockdown of circular RNA UBAP2 inhibits the malignant behaviours of esophageal squamous cell carcinoma by microRNA-422a/Rab10 axis. Clin Exp Pharmacol Physiol.

[89] Song H, Xu Y, Xu T, Fan R, Jiang T, Cao M (2020). CircPIP5K1A activates KRT80 and PI3K/AKT pathway to promote gastric cancer development through sponging miR-671-5p. Biomed Pharmacother.

[90] Han K, Wang FW, Cao CH, Ling H, Chen JW, Chen RX (2020). CircLONP2 enhances colorectal carcinoma invasion and metastasis through modulating the maturation and exosomal dissemination of microRNA-17. Mol Cancer.

[91] Geng Y, Zheng X, Hu W, Wang Q, Xu Y, He W (2019). Hsa_circ_0009361 acts as the sponge of miR-582 to suppress colorectal cancer progression by regulating APC2 expression. Clin Sci (Lond).

[92] Xie H, Ren X, Xin S, Lan X, Lu G, Lin Y (2016). Emerging roles of circRNA_001569 targeting miR-145 in the proliferation and invasion of colorectal cancer. Oncotarget.

[93] Xie B, Zhao Z, Liu Q, Wang X, Ma Z, Li H (2019). CircRNA has_circ_0078710 acts as the sponge of microRNA-31 involved in hepatocellular carcinoma progression. Gene.

[94] Wang L, Long H, Zheng Q, Bo X, Xiao X, Li B (2019). Circular RNA circRHOT1 promotes hepatocellular carcinoma progression by initiation of NR2F6 expression. Mol Cancer.

[95] Sun S, Wang W, Luo X, Li Y, Liu B, Li X (2019). Circular RNA circ-ADD3 inhibits hepatocellular carcinoma metastasis through facilitating EZH2 degradation via CDK1-mediated ubiquitination. Am J Cancer Res.

[96] Zhang Z, Li X, Xiong F, Ren Z, Han Y (2020). Hsa_circ_0012563 promotes migration and invasion of esophageal squamous cell carcinoma by regulating XRCC1/EMT pathway. J Clin Lab Anal.

[97] Zhou LH, Yang YC, Zhang RY, Wang P, Pang MH, Liang LQ (2018). CircRNA_0023642 promotes migration and invasion of gastric cancer cells by regulating EMT. Eur Rev Med Pharmacol Sci.

[98] Pan Z, Cai J, Lin J, Zhou H, Peng J, Liang J (2020). A novel protein encoded by circFNDC3B inhibits tumor progression and EMT through regulating Snail in colon cancer. Mol Cancer.

[99] Li YF, Pei FL, Cao MZ (2020). CircRNA_101951 promotes migration and invasion of colorectal cancer cells by regulating the KIF3A-mediated EMT pathway. Exp Ther Med.

[100] Huang XY, Huang ZL, Huang J, Xu B, Huang XY, Xu YH (2020). Exosomal circRNA-100338 promotes hepatocellular carcinoma metastasis via enhancing invasiveness and angiogenesis. J Exp Clin Cancer Res.

[101] Pu J, Wang J, Li W, Lu Y, Wu X, Long X (2020). hsa_circ_0000092 promotes hepatocellular carcinoma progression through up-regulating HN1 expression by binding to microRNA-338-3p. J Cell Mol Med.

[102] Zhang Y, Li C, Liu X, Wang Y, Zhao R, Yang Y (2019). circHIPK3 promotes oxaliplatin-resistance in colorectal cancer through autophagy by sponging miR-637. EBioMedicine.

[103] Lai M, Liu G, Li R, Bai H, Zhao J, Xiao P (2020). Hsa_circ_0079662 induces the resistance mechanism of the chemotherapy drug oxaliplatin through the TNF-α pathway in human colon cancer. J Cell Mol Med.

[104] Luo Y, Fu Y, Huang R, Gao M, Liu F, Gui R (2019). CircRNA_101505 sensitizes hepatocellular carcinoma cells to cisplatin by sponging miR-103 and promotes oxidored-nitro domain-containing protein 1 expression. Cell Death Discov.

[105] Qiao GL, Chen L, Jiang WH, Yang C, Yang CM, Song LN (2019). Hsa_circ_0003998 may be used as a new biomarker for the diagnosis and prognosis of hepatocellular carcinoma. Onco Targets Ther.

[106] Yang F, Liu DY, Guo JT, Ge N, Zhu P, Liu X (2017). Circular RNA circ-LDLRAD3 as a biomarker in diagnosis of pancreatic cancer. World J Gastroenterol.

[107] Lu J, Zhang PY, Xie JW, Wang JB, Lin JX, Chen QY (2019). Circular RNA hsa_circ_0006848 related to ribosomal protein L6 acts as a novel biomarker for early gastric cancer. Dis Markers.

[108] Li Z, Zhou Y, Yang G, He S, Qiu X, Zhang L (2019). Using circular RNA SMARCA5 as a potential novel biomarker for hepatocellular carcinoma. Clin Chim Acta.

[109] Fan L, Cao Q, Liu J, Zhang J, Li B (2019). Circular RNA profiling and its potential for esophageal squamous cell cancer diagnosis and prognosis. Mol Cancer.

[110] Hu K, Qin X, Shao Y, Zhou Y, Ye G, Xu S (2020). Circular RNA MTO1 suppresses tumorigenesis of gastric carcinoma by sponging miR-3200-5p and targeting PEBP1. Mol Cell Probes.

[111] Yu J, Ding WB, Wang MC, Guo XG, Xu J, Xu QG (2020). Plasma circular RNA panel to diagnose hepatitis B virus-related hepatocellular carcinoma: a large-scale, multicenter study. Int J Cancer.

[112] Lin J, Cai D, Li W, Yu T, Mao H, Jiang S (2019). Plasma circular RNA panel acts as a novel diagnostic biomarker for colorectal cancer. Clin Biochem.

[113] Bai H, Lei K, Huang F, Jiang Z, Zhou X (2019). Exo-circRNAs: a new paradigm for anticancer therapy. Mol Cancer.

[114] Wang Y, Liu J, Ma J, Sun T, Zhou Q, Wang W (2019). Exosomal circRNAs: biogenesis, effect and application in human diseases. Mol Cancer.

[115] Louis C, Desoteux M, Coulouarn C (2019). Exosomal circRNAs: new players in the field of cholangiocarcinoma. Clin Sci (Lond).

[116] Shao Y, Tao X, Lu R, Zhang H, Ge J, Xiao B (2020). Hsa_circ_0065149 is an Indicator for early gastric cancer screening and prognosis prediction. Pathol Oncol Res.

[117] Shao Y, Li J, Lu R, Li T, Yang Y, Xiao B (2017). Global circular RNA expression profile of human gastric cancer and its clinical significance. Cancer Med.

[118] Jiang XM, Li ZL, Li JL, Xu Y, Leng KM, Cui YF (2018). A novel prognostic biomarker for cholangiocarcinoma: circRNA Cdr1as. Eur Rev Med Pharmacol Sci.

[119] Su Y, Lv X, Yin W, Zhou L, Hu Y, Zhou A (2019). CircRNA Cdr1as functions as a competitive endogenous RNA to promote hepatocellular carcinoma progression. Aging (Albany NY).

[120] Tang W, Ji M, He G, Yang L, Niu Z, Jian M (2017). Silencing CDR1as inhibits colorectal cancer progression through regulating microRNA-7. Onco Targets Ther.

[121] Chen J, Li Y, Zheng Q, Bao C, He J, Chen B (2017). Circular RNA profile identifies circPVT1 as a proliferative factor and prognostic marker in gastric cancer. Cancer Lett.

[122] Cao S, Chen G, Yan L, Li L, Huang X (2018). Contribution of dysregulated circRNA_100876 to proliferation and metastasis of esophageal squamous cell carcinoma. Onco Targets Ther.

[123] Huang E, Fu J, Yu Q, Xie P, Yang Z, Ji H (2020). CircRNA hsa_circ_0004771 promotes esophageal squamous cell cancer progression via miR-339-5p/CDC25A axis. Epigenomics.

[124] Zhang J, Liu H, Hou L, Wang G, Zhang R, Huang Y (2017). Circular RNA_LARP4 inhibits cell proliferation and invasion of gastric cancer by sponging miR-424-5p and regulating LATS1 expression. Mol Cancer.

[125] Liu H, Liu Y, Bian Z, Zhang J, Zhang R, Chen X (2018). Circular RNA YAP1 inhibits the proliferation and invasion of gastric cancer cells by regulating the miR-367-5p/p27 (Kip1) axis. Mol Cancer.

[126] Han D, Li J, Wang H, Su X, Hou J, Gu Y (2017). Circular RNA circMTO1 acts as the sponge of microRNA-9 to suppress hepatocellular carcinoma progression. Hepatology.

[127] Bian L, Zhi X, Ma L, Zhang J, Chen P, Sun S (2018). Hsa_circRNA_103809 regulated the cell proliferation and migration in colorectal cancer via miR-532-3p/FOXO4 axis. Biochem Biophys Res Commun.

[128] Zhang W, Yang S, Liu Y, Wang Y, Lin T, Li Y (2018). Hsa_circ_0007534 as a blood-based marker for the diagnosis of colorectal cancer and its prognostic value. Int J Clin Exp Pathol.

[129] Wang Q, Liu H, Liu Z, Yang L, Zhou J, Cao X (2020). Circ-SLC7A5, a potential prognostic circulating biomarker for detection of ESCC. Cancer Genet.

[130] Ba Y, Liu Y, Li C, Zhu Y, Xing W (2020). HIPK3 Promotes growth and metastasis of esophageal squamous cell carcinoma via regulation of miR-599/c-MYC Axis. Onco Targets Ther.

[131] Wei J, Xu H, Wei W, Wang Z, Zhang Q, De W (2020). circHIPK3 promotes cell proliferation and migration of gastric cancer by sponging miR-107 and regulating BDNF expression. Onco Targets Ther.

[132] Yan Y, Su M, Qin B (2020). CircHIPK3 promotes colorectal cancer cells proliferation and metastasis via modulating of miR-1207-5p/FMNL2 signal. Biochem Biophys Res Commun.

[133] Liu WG, Xu Q (2019). Upregulation of circHIPK3 promotes the progression of gastric cancer via Wnt/β-catenin pathway and indicates a poor prognosis. Eur Rev Med Pharmacol Sci.

[134] Li J, Qin X, Wu R, Wan L, Zhang L, Liu R (2020). Circular RNA circFBXO11 modulates hepatocellular carcinoma progress and oxaliplatin resistance through miR-605/FOXO3/ABCB1 axis. J Cell Mol Med.

[135] Wang Z, Zhao Y, Wang Y, Jin C (2019). Circular RNA circHIAT1 inhibits cell growth in hepatocellular carcinoma by regulating miR-3171/PTEN axis. Biomed Pharmacother.

[136] Rong D, Lu C, Zhang B, Fu K, Zhao S, Tang W (2019). CircPSMC3 suppresses the proliferation and metastasis of gastric cancer by acting as a competitive endogenous RNA through sponging miR-296-5p. Mol Cancer.

[137] Gong R, Jiang Y (2020). Non-coding RNAs in pancreatic ductal adenocarcinoma. Front Oncol.

[138] Xing Y, Zha WJ, Li XM, Li H, Gao F, Ye T (2020). Circular RNA circ-Foxo3 inhibits esophageal squamous cell cancer progression via the miR-23a/PTEN axis. J Cell Biochem.

[139] Fu X, Zhang J, He X, Yan X, Wei J, Huang M (2020). Circular RNA MAN2B2 promotes cell proliferation of hepatocellular carcinoma cells via the miRNA-217/MAPK1 axis. J Cancer.

[140] Li Z, Liu Y, Yan J, Zeng Q, Hu Y, Wang H (2020). Circular RNA hsa_circ_0056836 functions an oncogenic gene in hepatocellular carcinoma through modulating miR-766-3p/FOSL2 axis. Aging (Albany NY).

[141] Wang L, Shen J, Jiang Y (2018). Circ_0027599/PHDLA1 suppresses gastric cancer progression by sponging miR-101-3p.1. Cell Biosci.

[142] Jiang Y, Wang T, Yan L, Qu L (2018). A novel prognostic biomarker for pancreatic ductal adenocarcinoma: hsa_circ_0001649. Gene.

[143] Nie H, Wang YT, Liao ZM, Zhou JH, Ou CL (2020). The function and mechanism of circular RNAs in gastrointestinal tumours. Cell Prolif.

[144] Naeli P, Pourhanifeh MH, Karimzadeh MR, Shabaninejad Z, Movahedpour A (2020). Circular RNAs and gastrointestinal cancers: epigenetic regulators with a prognostic and therapeutic role. Crit Rev Oncol Hematol.

